# Stabilizing Layered Structure in Aqueous Electrolyte via O2‐Type Oxygen Stacking

**DOI:** 10.1002/advs.202202194

**Published:** 2022-07-26

**Authors:** Liang Xue, Chao Wang, Hanghui Liu, Hao Li, Tingting Chen, Zhengyi Shi, Ce Qiu, Mingqing Sun, Yin Huang, Jiangfeng Huang, Jingwen Sun, Pan Xiong, Junwu Zhu, Hui Xia

**Affiliations:** ^1^ Key Laboratory for Soft Chemistry and Functional Materials of Ministry of Education Nanjing University of Science and Technology Nanjing 210094 China; ^2^ School of Materials Science and Engineering Nanjing University of Science and Technology Nanjing 210094 China

**Keywords:** lithium‐ion batteries, cathode materials, layered structure, O2 stacking, aqueous electrolyte

## Abstract

Despite the high energy density of O3‐type layered cathode materials, the short cycle life in aqueous electrolyte hinders their practical applications in aqueous lithium‐ion batteries (ALIBs). In this work, it is demonstrated that the structural stability of layered LiCoO_2_ in aqueous electrolyte can be remarkably improved by altering the oxygen stacking from O3 to O2. As compared to the O3‐type LiCoO_2_, the O2‐type LiCoO_2_ exhibits significantly improved cycle performance in neutral aqueous electrolyte. It is found that the structural degradation caused by electrophilic attack of proton can be effectively mitigated in O2‐type layered structure. With O2 stacking, CoO_6_ octahedra in LiCoO_2_ possess stronger Co—O bonds while Co migration from Co layer to Li layer is strongly hampered, resulting in enhanced structural stability against proton attack and prolonged cycle life in aqueous electrolyte. The findings in this work reveal that regulating oxygen stacking sequence is an effective strategy to improve the structural stability of layered materials for ALIBs.

## Graphical abstract

By altering the oxygen stacking sequence from O3 to O2, the layered structure stability can be significantly improved in the 1 m Li_2_SO_4_ aqueous electrolyte. With stronger Co─O bonds, the O2‐LiCoO_2_ exhibits enhanced Co confinement in CoO_6_ octahedra. Meanwhile, the face‐sharing local environments of cations in O2‐LiCoO_2_ effectively suppress Co migration to Li layers, thus inhibiting the layered‐to‐spinel phase transition.

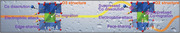

## 1 Introduction

The aqueous lithium‐ion batteries (ALIBs) have recently attracted intense attention owing to the intriguing merits of aqueous electrolytes including high safety, nonflammability, environmental benignity, high ionic mobility, and low cost.^[^ ^1^ ^]^ The narrow electrochemical window of water (1.23 V vs SHE), however, limits the selection of cathode and anode materials for ALIBs.^[^ ^2^ ^]^ In terms of cathode materials, the layered LiMO_2_ (M = Ni, Co, Mn, etc.) with suitably high electrode potential and large specific capacity are considered as promising cathode candidates for ALIBs. The O3‐type layered LiMO_2_ cathodes, where O represents Li^+^ occupied octahedral sites and the number 3 represents the oxygen stacking in “ABCABC” sequence, have achieved dramatic success in lithium‐ion batteries with organic electrolytes.^[^ ^3^ ^]^ However, these layered materials suffer from structural instability and rapid capacity decay upon cycling in aqueous electrolytes, which hampers the practical development of ALIBs.^[^ ^4^ ^]^ Recently, our group investigated the degradation mechanism of O3‐type LiCoO_2_ in aqueous electrolyte and found that the irreversible layered‐to‐spinel phase transition accelerated by proton attack is the main cause for structural degradation and fast capacity fading.^[^ ^5^ ^]^ Therefore, it is of great importance for ALIBs to develop strengthened layered structures that can suppress the layered‐to‐spinel phase transition during cycling.

The layered‐to‐spinel phase transition in O3‐type LiMO_2_ proceeds through the M migration from octahedral sites at M layer to octahedral sites at Li layer via tetrahedral intermediate sites. A recent work by Kang et al. demonstrated that by developing O2‐type lithium‐rich nickel manganese oxide with oxygen stacking in “BABCBA” sequence, the transition metal ions are difficult to migrate to the octahedral sites in the Li layer, which remarkably improves the reversibility of the transition metal ion migration and thereby dramatically enhances the structural stability during cycling.^[^ ^6^ ^]^ In O2‐type layered structure, the transition metal ion migration to the octahedral site in Li layer is demonstrated to be unfavorable due to the large electrostatic repulsion between face‐shared cations, which could effectively suppress the layered‐to‐spinel phase transition. It is therefore speculated that similar structural regulation strategy could be implemented for layered LiMO_2_ cathodes to improve their cycle performance in aqueous electrolyte. Nevertheless, no direct study has been reported for investigating the electrochemical properties of O2‐type LiMO_2_ in aqueous electrolytes by far.

In the present work, we synthesized O2‐type LiCoO_2_ (O2‐LCO) and explored its structural and electrochemical stability in neutral aqueous electrolyte (1 m Li_2_SO_4_) in comparison with O3‐type LiCoO_2_ (O3‐LCO). It has been demonstrated that significantly improved cycle performance can be achieved in aqueous electrolyte for LiCoO_2_ via altering the oxygen stacking sequence from O3 to O2. Through structural characterizations, we disclosed that the O2‐LCO possesses stronger Co─O bonds than O3‐LCO, which can help O2‐LCO resist the electrophilic attack of H^+^ on lattice oxygen. Meanwhile, the face‐sharing local environments of cations in O2‐LCO can effectively suppress Co migration to Li layers, thus inhibiting the layered‐to‐spinel phase transition. As a result, the O2‐LCO exhibits remarkably enhanced structural stability and greatly attenuated electrochemical degradation as compared to the O3‐LCO in aqueous electrolyte. The demonstration of enhanced structure stability of O2‐type layered material in aqueous electrolyte provides valuable insights in structural regulation to develop high performance cathodes for ALIBs.

## 2 Results and Discussion

### 2.1 Electrochemical Performance

**Figure 1:**
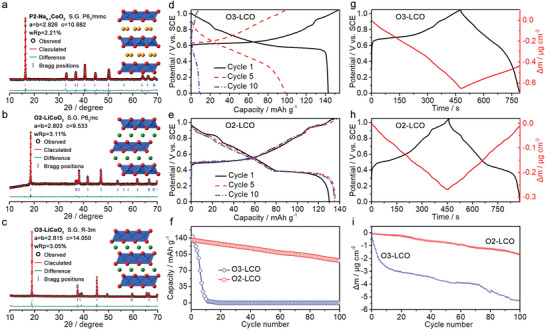
XRD patterns and Rietveld‐refined results of a) P2‐NCO, b) O2‐LCO, and c) O3‐LCO. Initial charge/discharge curves of the d) O3‐LCO and e) O2‐LCO electrodes between 0 and 1.05 V (vs SCE) at 1 C in 1 m Li_2_SO_4_ aqueous electrolyte. f) Cycle performance of the O3‐LCO and O2‐LCO electrodes at 1 C for 100 cycles. Electrode mass changes of the g) O3‐LCO and h) O2‐LCO electrodes between charge and discharge processes in aqueous electrolyte. i) Electrode mass changes of the O3‐LCO and O2‐LCO electrodes during cycling in aqueous electrolyte.

The O2‐LCO was synthesized from P2‐Na_0.7_CoO_2_ (P2‐NCO) by a hydrothermal ion‐exchange method. The X‐ray diffraction (XRD) patterns and Rietveld‐refined results in **Figure** **1**a,b illustrate the phase transformation from P2‐NCO (with space group of *P*6_3_ */mmc* and BAABBA oxygen stacking, Figure 1a) to O2‐LCO (with space group of *P*6_3_ *mc* and BABCBA oxygen stacking, Figure 1b). The P2 to O2 phase transition results from the gliding of CoO_2_ slabs in P2‐NCO, which is driven by the replacement of prismatic Na sites by octahedral Li sites.^[^ ^7^ ^]^ To investigate whether Na^+^ ions remained in O2‐LCO after hydrothermal Li/Na ion exchange reaction of P2‐NCO, we further performed solid‐state ^23^Na magic angle spinning (MAS) NMR on the O2‐LCO. As shown in Figure S1 (Supporting Information), the O2‐LCO exhibits a weak ^23^Na MAS NMR signal located around 435 ppm, which can be attributed to the Fermi contact shift induced by spin‐transfer from paramagnetic Co^4+^ to the prismatic Na^+^ ions in the residual of Na*_x_* CoO_2_ phase.^[^ ^8^ ^]^ Compared with prismatic Na^+^, octahedral Na^+^ ions have stronger Fermi contact with Co^4+^. However, we did not detect the signal of the octahedral Na^+^ ions at the low field,^[^ ^9^ ^]^ demonstrating no Na^+^ ions exist in O2‐LCO. Therefore, Na^+^ ions could exist in the trace amount of residual P2‐NCO phase but are unlikely to exist in the O2‐LCO after ion exchange. In addition, we carried out XRD measurements on both fresh O2‐LCO sample and old O2‐LCO sample stored in a plastic bag for nearly one year. As shown in Figure S2 (Supporting Information), the two XRD patterns are almost identical with all diffraction peaks overlapping, indicating that O2‐LCO is air‐stable. To test structure stability of O2‐LCO in aqueous solution, we put the as‐synthesized O2‐LCO powder in pure water for 24 h with continuous stirring. After that, the O2‐LCO powder was centrifuged, washed, and dried for XRD measurement. The XRD pattern and Rietveld‐refined results of O2‐LCO after water exposure (Figure S3, Supporting Information) demonstrate that the layered structure is well retained after water exposure without interlayer expansion. The O3‐LCO was synthesized by conventional solid‐state reaction and the XRD pattern and Rietveld‐refined results are presented in Figure 1c (with space group of *R‐*3*m* and ABCABC oxygen stacking). Owing to different oxygen stacking sequences, the local environments of Li sites in O2 and O3 are very different: LiO_6_ octahedra share both edges on one side and faces on the other side with CoO_6_ octahedra in O2 while LiO_6_ octahedra share edges with CoO_6_ octahedra in O3. Despite different layered structures, the O3‐LCO and O2‐LCO electrodes exhibit comparative specific capacities (≈135 mAh g^−1^ at 1 C) and similar cycle performance (≈88% capacity retention after 100 cycles) when cycled between 3 and 4.2 V (vs Li/Li^+^) in organic electrolyte (Figure S4, Supporting Information). However, the electrochemical behaviors of the O3‐LCO and O2‐LCO electrodes are notably different in 1 m Li_2_SO_4_ aqueous electrolyte. As shown in Figure 1d,e, the initial charge/discharge curves demonstrate that O2‐LCO has lower voltage plateaus and slightly smaller initial charge/discharge capacities than those of O3‐LCO. Although O3‐LCO shows a larger initial reversible capacity, it drops quickly to nearly zero only after 10 cycles with sharp increase in electrode polarization, revealing severe structural degradation during cycling. In comparison, O2‐LCO exhibits highly reversible charge/discharge processes during cycling as no capacity fading can be detected for the initial cycles, suggesting greatly improved structural stability. As compared in Figure 1f, the capacity retention of the O2‐LCO electrode is 72.7% after 100 cycles, which is remarkably higher than that of the O3‐LCO electrode (6.0% after 10 cycles), demonstrating significantly improved cycle performance of O2‐LCO in aqueous electrolyte. These results indicate that altering the oxygen stacking sequence in layered structure can viably improve its structural stability in aqueous electrolyte and O2 stacking could more effectively resist the proton attack.

To understand the difference in structural stability between O3‐LCO and O2‐LCO, we used the electrochemical quartz crystal microbalance (EQCM) measurements to monitor the electrode mass change of the O3‐LCO and O2‐LCO electrodes during charge/discharge processes in aqueous electrolyte. As shown in Figure 1g and Figure S5a (Supporting Information), the O3‐LCO electrode exhibits a mass loss with ∆*m*/*dq* of −19 g mol^−1^ during charge process and a mass gain with ∆*m*/*dq* of 8 g mol^−1^ during discharge process. The mass gain during discharge can be well attributed to Li^+^ intercalation while the much larger mass loss during charge could stem from a combination of Li^+^ deintercalation and Co dissolution. The unmatched mass changes between charge and discharge for the O3‐LCO electrode indicate its poor structural stability in aqueous electrolyte. In comparison, the O2‐LCO electrode exhibits a mass loss with ∆*m*/*dq* of −9 g mol^−1^ for charge process and a mass gain with ∆*m*/*dq* of 8 g mol^−1^ for discharge process (Figure 1h and Figure S5b, Supporting Information), suggesting nearly reversible Li^+^ deintercalation and intercalation in the layered structure matrix. It is noticed that the mass loss of charge is only slightly larger than that of discharge, revealing that Co dissolution into aqueous electrolyte during charge can be effectively suppressed in O2‐LCO. As shown in Figure 1i, further EQCM measurements along with cycling demonstrate that severe electrode mass loss continues in O3‐LCO electrode during cycling, which is consistent with the fast capacity fading in Figure 1f, suggesting rapid structural degradation associated with Co dissolution of O3 layered structure in aqueous electrolyte. The notably reduced electrode mass loss during cycling together with significantly improved cycle performance for O2‐LCO suggest greatly enhanced structural stability of layered transition metal oxide with O2 stacking in aqueous electrolyte.

### 2.2 Structural Evolution in Aqueous Electrolyte

**Figure 2:**
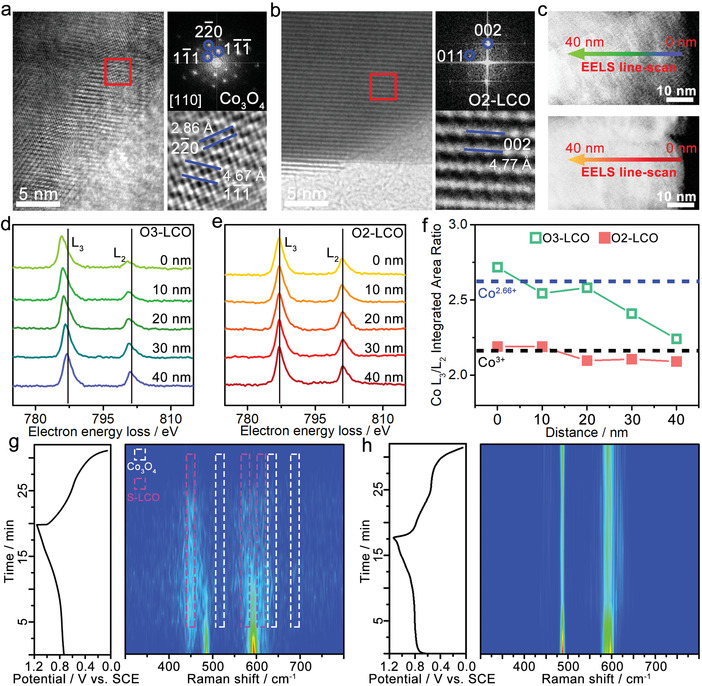
HRTEM images and corresponding FFT images of a) O3‐LCO and b) O2‐LCO after 100 cycles in aqueous electrolyte. c) HAADF‐STEM images with EELS line‐scan paths of O3‐LCO and O2‐LCO. Selected EELS Co L_3,2_‐edge spectra at different depths of d) O3‐LCO and e) O2‐LCO from surface to subsurface region. f) Co L_3_/L_2_ integrated intensity ratios at different depths for both O3‐LCO and O2‐LCO. In situ Raman spectra of g) O3‐LCO and h) O2‐LCO during the first charge/discharge processes in aqueous electrolyte.

To investigate structural evolutions of O3‐LCO and O2‐LCO in aqueous electrolyte during cycling, the two electrodes before and after 100 cycles were characterized by XRD and high‐resolution transmission electron microscope (HRTEM). The obtained XRD patterns of O3‐LCO and O2‐LCO electrodes after 100 cycles are presented in Figure S6 (Supporting Information). It is noted that obvious change can be detected from the XRD pattern of O3‐LCO after 100 cycles. The intensity ratio of 003 to 104 is obviously weakened, and the originally separated doublets of 006/012 and 018/110 merge into one peak, suggesting O3‐LCO undergoes the layered‐to‐spinel phase transition during cycling in aqueous electrolyte. In comparison, the O2‐LCO shows the negligible change in the XRD pattern after 100 cycles, indicating the O2‐type layered structure is well maintained after cycling in aqueous electrolyte. The HRTEM and corresponding FFT images are provided in Figure S7 (Supporting Information). Compared to the cycled samples in **Figure** **2** and Figure S8 (Supporting Information), the as‐prepared O3‐LCO and O2‐LCO samples show highly crystallized layered structure with clean surface. After 100 cycles, a ≈17 nm thick surface film with nanocracks is formed on the O3‐LCO particle (Figure S8a, Supporting Information), while the O2‐LCO particle retains the smooth and intact surface (Figure S8b, Supporting Information). Figure 2a shows the enlarged HRTEM image at the surface region of the cycled O3‐LCO, displaying distorted lattice fringes. The corresponding fast Fourier transform (FFT) pattern can be well indexed to the spinel Co_3_O_4_ and the reflection of (2−20) represents the Co occupancy at 8a tetrahedral sites in spinel. By contrast, the O2‐LCO maintains the well‐resolved layered structure at both surface and subsurface regions after cycling in aqueous electrolyte (Figure 2b). The lattice fringes and corresponding FFT pattern at the surface in Figure 2b can be well attributed to the layered structure, suggesting no obvious structural degradation of O2‐LCO after 100 cycles.

Electron energy loss spectroscopy (EELS) spectra of Co L_3,2_‐edges, corresponding to excitations of the 2p electrons to 3d orbitals, are sensitive to monitor the valence change of Co in O3‐LCO and O2‐LCO cycled in aqueous electrolyte. The EELS line‐scans were performed from surface to subsurface regions for both samples as indicated in high‐angle annular dark‐field scanning transmission electron microscopy (HAADF‐STEM) images in Figure 2c. The selected EELS Co L_3,2_‐edge spectra show red shift of both L_3_‐ and L_2_‐edges toward the surface for O3‐LCO (Figure 2d) while such shift is not detected for O2‐LCO (Figure 2e). The red shift with the energy loss generally indicates a decrease of Co valence state because screening of the added electrons leads to a higher energy level of the 2p orbital.^[^ ^10^ ^]^ A more quantitative probing of the Co valence state can be obtained by acquiring the L_3_/L_2_ integrated intensity ratio.^[^ ^11^ ^]^ As shown in Figure 2f, the L_3_/L_2_ ratio of O3‐LCO increases when moving toward the surface, suggesting reduction of Co valence at the surface. Based on the L_3_/L_2_ ratios of LCO (Co^3+^) and Co_3_O_4_ (Co^2.66+^) standards,^[^ ^12^ ^]^ the average oxidation state of Co for O3‐LCO near the surface is estimated to be 2.66+, agreeing well with the formation of spinel Co_3_O_4_ surface layer as indicated by HRTEM result. In comparison, the L_3_/L_2_ ratio of O2‐LCO remains nearly constant at ≈2.2, which corresponds to Co^3+^ in LCO, demonstrating its stable surface in aqueous electrolyte. In addition, the X‐ray photoelectron spectroscopy (XPS) depth profile analysis (Figure S9, Supporting Information) of the surface Li demonstrates severe Li^+^ loss at the surface region for O3‐LCO, which is in accordance with the formation of spinel Co_3_O_4_.

To investigate the redox reactions of cobalt ions of O3‐LCO and O2‐LCO during charge/discharge in aqueous electrolyte, we have performed ex situ XPS measurements. As shown in Figure S10 (Supporting Information), the Co 2p XPS spectra of both O3‐LCO and O2‐LCO exhibit main peaks at 781 eV (Co 2p_3/2_) and 796 eV (Co 2p_1/2_) with satellite peaks at 790 eV (Co 2p_3/2_) and 805 eV (Co 2p_1/2_). During charge process, the broadening of main peaks and decrease of satellite peaks can be attributed to the oxidation process of Co^3+^.^[^ ^13^ ^]^ During discharge process, the XPS spectra of O3‐LCO show Co^2+^ feature with the satellite peaks shifting towards the main peaks,^[^ ^14^ ^]^ suggesting the formation of Co_3_O_4_ impurity phase in O3‐LCO. In comparison, the XPS spectra of O2‐LCO during discharge only show narrowing of main peaks and increase of satellite peaks without peak shifting, demonstrating highly reversible Co^3+/4+^ redox reaction during charge/discharge processes.

The in situ Raman spectra were further used to investigate the structure evolutions of O3‐LCO and O2‐LCO during the initial charge and discharge processes. As shown in Figure 2g, in addition to the characteristic E_g_ and A_1g_ Raman bands for O3‐LCO, several new bands appear during the initial charge process, which can be attributed to the spinel LiCoO_2_ (S‐LCO) and Co_3_O_4_ phases, indicating the layered‐to‐spinel phase transition occurs even at the first charge process. The Raman spectra of the O3‐LCO after 10, 50, and 100 cycles of charge/discharge in Figure S11 (Supporting Information) indicate that the S‐LCO surface is first generated followed by conversion into spinel Co_3_O_4_ during subsequent charge/discharge processes. For O2‐LCO, the in situ Raman spectra in Figure 2h maintain the original Raman bands during the whole charge/discharge processes, demonstrating well retained layered structure and greatly improved structural stability. As shown in Figure S12a (Supporting Information), the initial 10 cycles of CV curves of the O3‐LCO electrode show dramatic shape change and rapid reduction of the enclosed area, indicating fast structural degradation. Unlike O3‐LCO, Figure S12b (Supporting Information) shows almost overlapped CV curves for the initial 10 cycles with well‐retained redox peaks and highly reversible feature, indicating greatly enhanced layered structure stability of the O2‐LCO. Figure S13 (Supporting Information) displays the electrochemical impedance spectroscopy (EIS) spectra of the O3‐LCO and O2‐LCO electrodes at the 1^st^, 50^th^, and 100^th^ cycles. It is noticed that the charge transfer resistance, corresponding to the semicircle, of the O3‐LCO electrode increases quickly with the cycle numbers, indicating the poor Li^+^ conductive nature of the formed surface spinel phase (S‐LCO and Co_3_O_4_). By contrast, the charge transfer resistance of the O2‐LCO electrode only displays minimal increase with cycling, demonstrating greatly enhanced surface stability in aqueous electrolyte. Based on the structural evolution analysis, we can see O3‐LCO is highly unstable when cycled in aqueous electrolyte, and its structural degradation proceeds through the layered‐to‐spinel phase transition associated with Co dissolution into the electrolyte. The formed spinel Co_3_O_4_ surface layer is electrochemically inactive and severely reduces the electrode kinetics, resulting in fast capacity fading in aqueous electrolyte. It's interesting to discover the remarkably enhanced structural stability and improved electrochemical performance of O2‐LCO in aqueous electrolyte. Although slight Co dissolution still persists in O2‐LCO, no layered‐to‐spinel phase transition can be detected on the O2‐LCO surface during cycling.

Although the O2‐LCO shows improved layered structure stability than O3‐LCO in aqueous electrolyte, its capacity retention after 100 cycles (72.7%) is smaller than that in organic electrolyte (≈88%). In addition to the surface structure evolution, the bulk phase transition mechanism of O2‐LCO in aqueous electrolyte and organic electrolyte needs to be further explored. We have performed in situ XRD on O2‐LCO electrodes in both aqueous electrolyte and carbonate‐based electrolyte as shown in Figure S14 (Supporting Information). In organic electrolyte, the O2‐LCO exhibits a phase transition process consistent with literature report.^[^ ^15^ ^]^ During the charge process, the O2_1_ phase (002 at 18.4°) transforms into the O2_2_ (002 at 17.7°) phase, and the O6 phase appears together with O2_2_ at high potential. (006 at 17.9°). Such phase transition is highly reversible during the subsequent discharge process. In the aqueous electrolyte, O2‐LCO undergoes the same phase transition from O2_1_ to O2_2_ during the charge process. However, the O6 phase appears earlier during the charge process and exists in a wider potential range than that in the organic electrolyte. Nevertheless, it returns to the O2_1_ phase at the end of the discharge, showing good reversibility of the phase transition. It is speculated that the difference of O6 phase formation during phase transition of O2‐LCO in aqueous and organic electrolytes is probably caused by protonation of the material in aqueous electrolyte.

### 2.3 Electrophilic Attack of H^+^ on Lattice O

**Figure 3:**
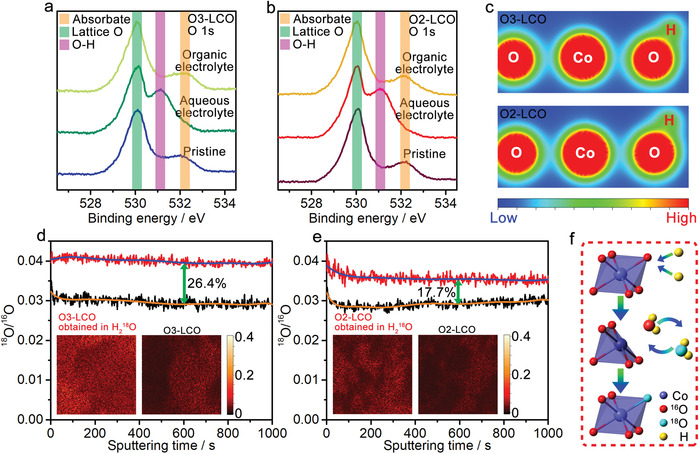
O 1s XPS spectra of a) O3‐LCO and b) O2‐LCO before and after one charge/discharge cycle in aqueous electrolyte and organic electrolyte. c) Contour maps of total charge density distributions in Co—O planes with one H—O bond of O3‐LCO and O2‐LCO. TOF‐SIMS ^18^O/^16^O isotopes frequency ratios as a function of specimen depth of d) O3‐LCO and e) O2‐LCO samples with (red curves) and without (black curves) the treatment in acid containing ^18^O‐enriched H_2_O. The insets are ^18^O/^16^O surface maps of sample with or without treatment in acid containing ^18^O‐enriched H_2_O. f) Schematic illustrations of oxygen elimination/addition mechanism for incorporating ^18^O in lattice.

As the structural stability of transition metal oxides is highly dependent on their interaction with H^+^ in aqueous electrolyte, it is important to further understand proton's influence on layered structures with different oxygen stackings. As revealed in recent studies, the H^+^ ions are electrophilic reagents and they may attack the lattice oxygen of the transition metal oxides.^[^ ^16^ ^]^ To investigate the interaction between proton and lattice oxygen, the O 1s XPS spectra of the O3‐LCO and O2‐LCO electrodes cycled in aqueous electrolyte and organic electrolyte are compared in **Figure** **3**a,b. For the O 1s XPS spectra of the pristine electrodes, a strong peak at ≈530 eV corresponds to lattice O while a minor peak at ≈532 eV can be assigned to weakly absorbed O species. Particularly, a peak at ≈531 eV for the cycled O3‐LCO and O2‐LCO electrodes in aqueous electrolytes can be attributed to lattice O—H bonds.^[^ ^17^ ^]^ The absence of the 531 eV peak in O 1s XPS spectra of the cycled electrodes in organic electrolyte further suggests the existence of proton attack on lattice oxygen for both two samples in aqueous electrolyte. To explore the influence of proton attack on the structural stability, the density functional theory (DFT) calculations have been carried out on the two‐layered structure models. Based on the calculated total charge density distributions in Co─O planes of O3‐LCO and O2‐LCO (Figure 3c), the formation of strong oxygen–hydrogen covalent bond decreases the electron cloud overlap between Co and O atoms, significantly weakening the Co—O bond strength. Nevertheless, the Co—O bond length adjacent to hydrogen in O2‐LCO (1.929 Å) is notably shorter than that in O3‐LCO (1.944 Å) (Figure S15, Supporting Information), and the electron cloud overlap in O2‐LCO is obviously larger than that in O3‐LCO, indicating that the weakening of Co—O bonds owing to proton attack can be effectively attenuated in O2‐LCO as compared to O3‐LCO.

It has been reported that the protonation of the transition metal oxide at the solid–liquid interface could snatch the lattice oxygen to form H_2_O.^[^ ^18^ ^]^ This protonation can induce a disproportionation reaction for Jahn‐Teller active oxides, such as LiMn_2_O_4_, which accelerates metal ion dissolution into the electrolyte. To further investigate the impact of protonation on lattice oxygen in O3‐LCO and O2‐LCO, we used ^18^O isotope labeling together with the time‐of‐flight secondary‐ion mass spectrometry (TOF‐SIMS). The TOF‐SIMS ^18^O/^16^O surface maps (insets in Figure 3d,e) reveal that both O3‐LCO and O2‐LCO show increased ^18^O content after being treated in an acidic solution containing ^18^O‐enriched H_2_O. Figure 3d,e shows the ^18^O/^16^O isotopes frequency ratio as a function of the specimen depth, in which the ^18^O content in the bulk of O3‐LCO is 8.7% higher than that in the bulk of O2‐LCO. The TOF‐SIMS results suggest that the electrophilic attack by H^+^ on the lattice ^16^O could lead to the formation of H_2_^16^O and accordingly the Co coordination changes from CoO_6_ to CoO_5_. Subsequently, CoO_5_ can further interact with H_2_^18^O to incorporate ^18^O into the lattice, which is schematically illustrated in Figure 3f. It is noticed that the concentrations of ^18^O in both O3 and O2‐LCO are much higher than that reported for LiMn_2_O_4_ with similar acid treatment, suggesting the disproportionation reaction could also occur in the Jahn‐Teller inactive oxides.^[^ ^19^ ^]^ With the formation of H_2_O in the protonated LCO, it is speculated that the electrophilic attack by H^+^ could drive the Co disproportionation reaction, which accelerates Co dissolution in aqueous electrolyte. Nevertheless, the relatively lower concentration of ^18^O in O2‐LCO than O3‐LCO demonstrates the improved resistivity to proton attack for the layered structure with O2 stacking, which is consistent with stronger Co—O bond with the formation of H—O bond in O2‐LCO.

### 2.4 Origin of Enhanced Structure Stability of O2 Stacking

**Figure 4:**
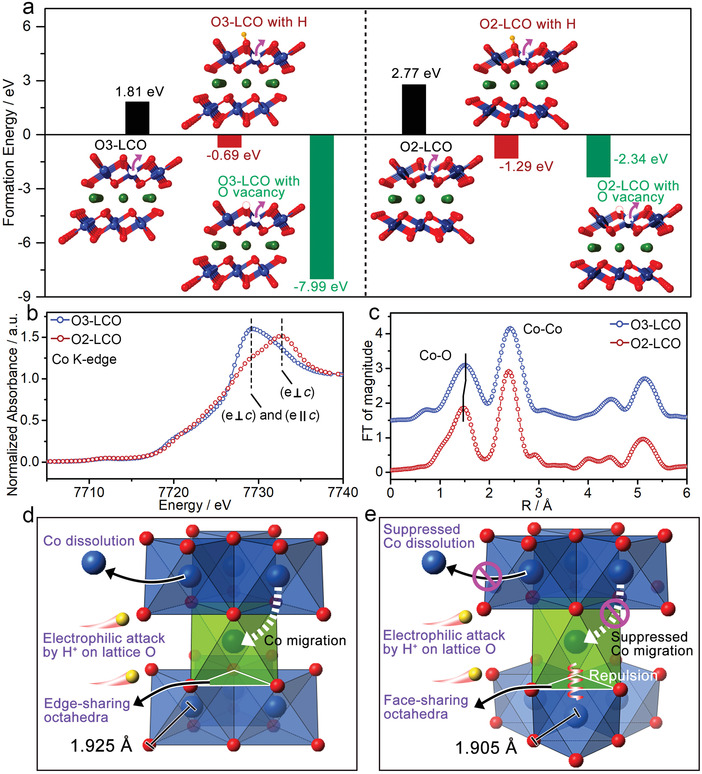
a) Co vacancy formation energies of O3‐LCO and O2‐LCO with and without H and O vacancies. b) Co K‐edge XANES spectra of O3‐LCO and O2‐LCO. c) Fourier transform of EXAFS spectra of O3‐LCO and O2‐LCO. Schematic illustrations of the crystal structures, electrophilic attack by H^+^ on the lattice oxygen, and Co migration paths of d) O3‐LCO and e) O2‐LCO.

Since both the layered‐to‐spinel phase transition and Co dissolution depend on the capability of Co migration out of the CoO_6_ octahedra, we calculated the Co vacancy formation energies *E*_f_ *(V*_Co_) for O3‐LCO and O2‐LCO with and without H^+^ attack by using DFT to evaluate the Co migration capability in these circumstances. Specifically, *E*_f_ *(V*_Co_) can be defined as follows:

(1) $$E_{f}\left(V_{\mathrm{Co}}\right)=E_{a}-E_{b}-\sum n_{i}\mu_{i}$$

where *E*_a_ and *E*_b_ denote the total energies of the structures with and without Co vacancy, respectively. *n_i_* denotes the number of element *i* that have been incorporated into (*n_i_* >0) or removed from (*n_i_* <0) the b structure. *µ_i_* denotes the chemical potential of element *i*. As shown in **Figure** **4**a, the Co vacancy formation energies in O3‐LCO and O2‐LCO supercells are +1.81 and +2.77 eV, respectively, which indicates that the Co vacancy formation is thermodynamically unfavorable in both layered structures without proton attack. However, the introduction of hydrogen to the layered structures can remarkably reduce the Co vacancy formation energies to negative values of −0.69 and −1.29 eV for O3‐LCO and O2‐LCO, respectively, suggesting Co vacancy formation becomes thermodynamically favorable with the assistance of hydrogen. In other words, the proton attack on lattice oxygen could promote the migration of Co in layered structures. In case of H_2_O formation with proton attack, an oxygen vacancy will be created in the CoO_6_ octahedron. The Co vacancy formation energies for O3‐LCO and O2‐LCO supercells with adjacent lattice O vacancy are −7.99 and −2.34 eV, respectively, indicating more favorable Co vacancy formation with the assistance of O vacancy. Under this circumstance, the O3‐LCO has higher probability for Co migration than O2‐LCO, further demonstrating enhanced structural stability of O2‐LCO. The Co vacancy formation energies in the half‐delithiated phases are calculated and presented in Figure S16 (Supporting Information). The Co vacancy formation energies for O3‐LCO and O2‐LCO supercells with adjacent lattice O vacancy at half delithiated state are −0.2 and +0.54 eV, respectively, suggesting more favorable Co vacancy formation in O3‐LCO. Therefore, for both lithiated and delithiatied states, O2‐LCO possesses improved structural stability in comparison to O3‐LCO based on the Co vacancy formation probability. Compared to the fully lithiated phases in Figure 4a, the half‐delithiated phases show higher Co vacancy formation energies, which is caused by stronger Co—O hybridization in the half‐delithiated phases.^[^ ^20^ ^]^

DFT calculations suggest O2‐LCO possesses reinforced CoO_6_ octahedra that can impede Co migration. To further analyze the structural difference between O3‐LCO and O2‐LCO, the Co K‐edge X‐ray absorption near edge structure (XANES) spectra of two samples were measured and are shown in Figure 4b. A clear difference can be observed in Co K‐edge XANES spectra for the two samples, especially for the absorption peaks near 7730 eV. As the O3‐LCO and O2‐LCO are layered materials, the electric dipole transition probability from the 1s core level at the Co K‐edge is determined by the polarization direction of the X‐ray. In principle, the direction of the electric field *e* to the *c* axis is either parallel (*e*∥*c*) or perpendicular (*e*⊥*c*). According to previous works in literature, the absorption peak at ≈7728 eV could be attributed to both (*e*∥*c*) and (*e*⊥*c*) transitions, while the peak at ≈7733 eV could be mainly attributed to the (*e*⊥*c*) transition.^[^ ^21^ ^]^ Due to the different oxygen stacking sequences, the O3‐LCO with transition probability from the (*e*∥*c*) transition shows increased absorption intensity at ≈7728 eV in comparison to that for O2‐LCO. The Fourier transforms of Co extended X‐ray absorption fine structure (EXAFS) spectra in Figure 4c show similar features for the first Co—O and second Co—Co coordination shells for both O3‐LCO and O2‐LCO. However, the Co—O bond lengths of O2‐LCO (1.905 Å) are notably shorter than that of O3‐LCO (1.925 Å). The Co—O bond lengths of O3‐LCO and O2‐LCO were also evaluated from XRD refinement, and the results are presented in Figure S17 (Supporting Information) in comparison with EXAFS result. XRD refinement reveals Co—O bond lengths of 1.919 Å and 1.868 Å for O3‐LCO and O2‐LCO, respectively, showing the same trend for Co—O bond length variation as the result from EXAFS. Since the light source wavelength of EXAFS (0.4354 Å) is much smaller than that of XRD (15.4 Å), EXAFS is supposed to be a more precise technique for fine structure analysis than normal XRD. Therefore, we mainly used the EXAFS data (Table S1, Supporting Information) for Co—O bond length comparison between the two samples in this work. The thermal contribution to Debye‐Waller factors (*σ* ^2^) has been reported as a practical measure of local bond strength.^[^ ^22^ ^]^ As shown in Table S1 (Supporting Information), the *σ* ^2^ of Co—O bonds for O3‐LCO and O2‐LCO are 0.32 and 0.28, respectively, demonstrating stronger Co─O bond strength of O2‐LCO than O3‐LCO. The stronger Co─O bond strength of O2‐LCO indicates increased energy barrier for Co migration, agreeing well with its improved structural stability in aqueous electrolyte.

Based on the above analyses, the distinct difference in structural stability of O3‐LCO and O2‐LCO in aqueous electrolyte originates from the different Co—O bond strengths and oxygen stacking sequences for two layered structures. The O3 and O2 layered structures with different structural features are illustrated in Figure 4d,e. When the two layered structures are exposed to aqueous electrolyte, they both are vulnerable to proton attack and the O—H bonds can be easily formed especially at the surface region. The formation of O—H bonds in the layered structures can weaken the neighboring Co—O bonds, which leads to increased probability for Co migration or Co dissolution, resulting in reduced structural stability during cycling. Particularly, when the lattice oxygen is taken away by proton attack, the probability for Co migration and dissolution could be remarkably increased. The confinement of Co in the CoO_6_ octahedra highly depends on the Co—O bond strength. As compared in Figure 4d,e, the average Co—O bond length of O2‐LCO is much shorter than that of O3‐LCO, which enables O2‐LCO with high resistivity against proton attack and greatly reduced mobility for Co. It is known that the layered‐to‐spinel phase transition is driven by Co migration from octahedral site in Co layer to the octahedral site in Li layer. In addition to the stronger Co—O bonds, the local face‐sharing configuration between LiO_6_ and CoO_6_ octahedra makes the migration of Co to Li site unfavorable because of the large electrostatic repulsion between face‐shared cations.^[^ ^6^ ^]^ But for O3‐LCO, the edge‐sharing configuration between LiO_6_ and CoO_6_ octahedra affords easier path for Co migration, thus favoring the layered‐to‐spinel phase transition. Owing to the beneficial structural features, the O2‐LCO exhibits superior structural stability to O3‐LCO in aqueous electrolyte, resulting in prominent extension of cycle life for layered materials in ALIBs.

## 3 Conclusions

In summary, we demonstrate the disparate structural stabilities of layered lithium cobalt oxide polymorphs of O3‐LCO and O2‐LCO in aqueous electrolyte. The O3‐LCO exhibits fast capacity fading due to the instant layered‐to‐spinel phase transition initiated at the particle surface associated with Co dissolution during cycling. In contrast, the O2‐LCO well maintains its layered structure with suppressed Co dissolution during cycling, leading to notable extension of cycle life in aqueous electrolyte. It is demonstrated that protonation occurs in both layered structures when cycled in aqueous electrolyte, and the formation of H—O bond could weaken the neighboring Co—O bond, increasing structural instability with enhanced Co migration probability. With shortened Co—O bond length, the O2‐LCO exhibits greatly improved resistibility to the electrophilic proton attack and enhanced Co confinement capability in octahedra. By altering the oxygen stacking sequence, face‐sharing local configuration between cations can be engineered to further impede Co migration to Li layers, effectively suppressing the layered‐to‐spinel phase transition. The present work provides new opportunities for designing layered cathode materials with both high energy density and long cycle life for ALIBs.

## 4 Experimental Section

### Materials Synthesis

The P2‐NCO was prepared by heating a mixture of Na_2_CO_3_ and Co_3_O_4_ (with Na/Co ratio of 0.7) at 850 °C for 12 h in air. The O2‐LCO was prepared by a hydrothermal ion exchange reaction. Typically, 1 g P2‐NCO was added into a 40 mL of LiCl (2.5 m) and LiOH (2.5 m) solution by continuous stirring. The solution was then sealed in a Teflon‐lined stainless steel autoclave and heated at 120 °C for 3 h. The O3‐LCO was prepared by heating a mixture of Li_2_CO_3_ and Co_3_O_4_ (with Li/Co ratio of 1.01) at 900 °C for 12 h in air.

### Materials Characterization

XRD (Bruker‐AXS D8 Advance, Cu K*α* radiation) was used to investigate the crystal structures of different samples. For the in situ XRD measurements in organic electrolyte, an in situ XRD cell (LIB‐XRD) with a Be window was used. The O2‐LCO film (was made by coating a slurry of 80 wt% active material, 10 wt% acetylene black, and 10 wt% polyvinylidene fluoride in *N*‐methylpyrrolidone on a smooth surface, dried at 60 °C for 12 h, and peeled off) and Li metal were used as cathode and anode, respectively. For the in situ XRD measurements in aqueous electrolyte, an in situ XRD cell (WB‐XRD) with a Kapton tape sealed window was used. The O2‐LCO electrode and active carbon electrode (were made by coating a slurry of 80 wt% active material, 10 wt% acetylene black, and 10 wt% polyvinylidene fluoride in N‐methylpyrrolidone on carbon cloth and dried at 60 °C for 12 h) were used as cathode and anode, respectively. The galvanostatic charge/discharge measurements were carried out on a potentiostat (Ivium Vertex) at a current density of 0.1 A g^−1^. The XRD Rietveld refinement analysis was carried out by using the GSAS program.^[^ ^23^ ^]^ The solid‐state ^23^Na MAS NMR was recorded on Agilent 600 DD2 spectrometer (Agilent, USA, magnetic field strength 14.1 T) at 158.64 MHz. The powder samples were placed in a pencil‐type zirconia rotor of 4.0 mm o.d. The spectra were obtained at a spinning speed of 10 kHz. The Na signal of tetramethylsilane (TMS) at 0 ppm was used as the reference of ^23^Na chemical shift. XPS (ESCALAB250Xi, ThermoFisher Scientific) was applied to investigate the surface composition changes of different samples. HRTEM (JEOL JEM‐2100F) and Raman (JASCO NRS‐1000DT, 532 nm, 50 mW) were used to investigate the structural evolutions of different samples. For STEM‐EELS measurements, the beam energy was 200 kV and the resolution was about 1 eV. For isotope labeling, 100 mg of prepared powders (O2‐LCO or O3‐LCO) were added into a 1 m H_2_SO_4_ solution (prepared with pure water and 19.6 vol% ^18^O‐enriched water, respectively) and stirred at 25°C for 12 h. The obtained samples were centrifuged and washed several times by distilled water and dried at 60°C for 12 h. The TOF‐SIMS (C‐TOF TESCAN, 59 pA, 5 kV) was employed for the analyses of ^18^O and ^16^O ions. The analyzed areas for depth profiling and high‐resolution mapping are typically 10 µm × 10 µm with the pixel of 512 × 512. For the in situ Raman measurements, a three‐electrode in situ Raman cell (EC‐RAIR‐H) with a thin quartz window (thickness, <0.5 mm) was used. The as‐prepared cathode (was placed at the top of the glassy carbon electrode nearing the quartz window), Pt wire, and Ag/AgCl electrode were used as working electrode, counter electrode, and reference electrode, respectively. The galvanostatic charge/discharge measurements were carried out on a potentiostat (Ivium Vertex) at a current density of 1 A g^−1^. The power of laser beam delivered to the electrode surface was ≈10% of the maximum 50 mW laser to avoid its damage to the sample. The acquisition time was 10 s with no accumulation and the frequency range was selected from 200 to 1800 cm^−1^. The spectral resolution of Raman spectra in the study was ca. 1.0 cm^−1^. The Co K‐edge XAFS spectra were carried out at the 44A beamline of National Synchrotron Radiation Research Center (NSRRC). The samples were ground and uniformly daubed on the special adhesive tape. The data were collected in fluorescence mode using a Lytle detector. The obtained EXAFS data were processed using the ATHENA module in the Demeter software packages. The post‐edge background was subtracted from the overall absorption and then normalized with respect to the edge‐jump step. To quantify the atomic structure parameters, least‐squares curve parameter fitting was performed using the ARTEMIS module of Demeter software packages.

### Electrochemical Measurements

The obtained O3‐LCO and O2‐LCO were used as active materials to prepare electrodes by using carbon cloth as current collector. Typically, the active material, acetylene black, and polyvinylidene fluoride in 8:1:1 mass ratio were mixed in N‐methylpyrrolidone to form a slurry. Then, the slurry was coated on carbon cloth and dried in a vacuum at 100 °C for 24 h. The mass loadings of the active materials were kept in the range of 3–4 mg cm^−2^. The electrochemical measurements such as EIS, CV, and charge/discharge tests were carried out by an electrochemical workstation (Bio‐Logic VMP3) using the three‐electrode cell, in which the prepared cathodes were used as the working electrodes, a carbon cloth supported active carbon (with mass loading of ≈12 mg cm^−2^) was used as the counter electrode, and SCE was used as the reference electrode. 1 m Li_2_SO_4_ solutions with pH values of 7 and 11 (adjusted by adding LiOH) were used as aqueous electrolytes. The EQCM (QCM200, Stanford Research Systems) measurements were applied to determine the mass change (∆*m*) of the electrode accompanied with charge/discharge tests. The exchanged molecular weight of ion (*M*_w_) was calculated by $M_{w}=\frac{\Delta m\;n\;F}{\Delta Q}$, where *F* is Faraday constant (96 485 C mol^−1^), *n* is the valence number of ions, and *∆Q* is the charge quantity passed through the electrode in Coulombs.

### DFT Calculations

All first‐principles calculations within the framework of DFT were performed using the Vienna Ab Initio Simulation Package (VASP).^[^ ^24^ ^]^ The generalized‐gradient approximation (GGA) in the form of Perdew‐Burke‐Ernzerhof (PBE)^[^ ^25^ ^]^ describes the exchange‐correction energy. The projector‐augmented wave (PAW) pseudopotential^[^ ^26^ ^]^ was chosen to describe the electron‐ion interaction. To correct the self‐interaction of Co 3*d* electrons, the Hubbard U of 4.91 eV was used, in line with previous literature.^[^ ^27^ ^]^ The cutoff energy was set to 650 eV. The 4×4×2 and 3×3×1 girds of *k*‐points sampling were adopted for bulk and surface calculations, respectively. All atoms were optimized to their equilibrium positions under a force convergence of 0.03 eV Å^−1^. Spin‐polarized calculations were performed for all calculations.

## Conflict of Interest

The authors declare no conflict of interest.

## Supporting information

Supporting Information

## Data Availability

The data that support the findings of this study are available from the corresponding author upon reasonable request.
