# Supplementary material for: Stabilizing Layered Structure in Aqueous Electrolyte via O2‐Type Oxygen Stacking
Source: Adv Sci (Weinh). 2022 Jul 26;9(27):2202194. doi: 10.1002/advs.202202194 (PMC9507384; doi:10.1002/advs.202202194)
Supplement: Supplementary file 1 — Supporting Information [file ADVS-9-2202194-s001.pdf]

Supporting Information

Stabilizing layered structure in aqueous electrolyte via O2-type oxygen stacking

Liang Xue, Chao Wang, Hanghui Liu, Hao Li, Tingting Chen, Zhengyi Shi, Ce Qiu, Mingqing Sun, Yin Huang, Jiangfeng Huang, Jingwen Sun, Pan Xiong, Junwu Zhu, Hui Xia**

L. Xue, C. Wang, Y. Huang, J. Huang, J. Sun, P. Xiong, J. Zhu, H. Xia

Key Laboratory for Soft Chemistry and Functional Materials of Ministry of Education,

Nanjing University of Science and Technology, Nanjing 210094, China

E-mail: zhujw@njust.edu.cn, xiahui@njust.edu.cn

H. Liu, H. Li, T. Chen, Z. Shi, C. Qiu, M. Sun, H. Xia

School of Materials Science and Engineering, Nanjing University of Science and Technology, Nanjing 210094, China

E-mail: xiahui@njust.edu.cn

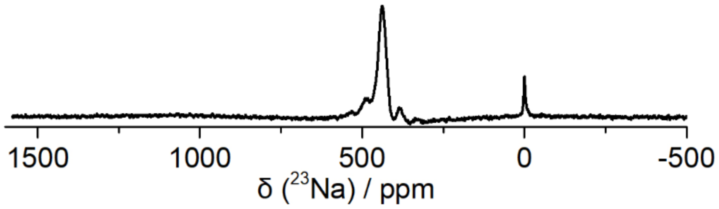

Figure S1. Solid-state ^{23}Na MAS NMR spectrum of synthesized O2-LCO. The sharp peak located around 0 ppm is attributed to diamagnetic sodium impurities.

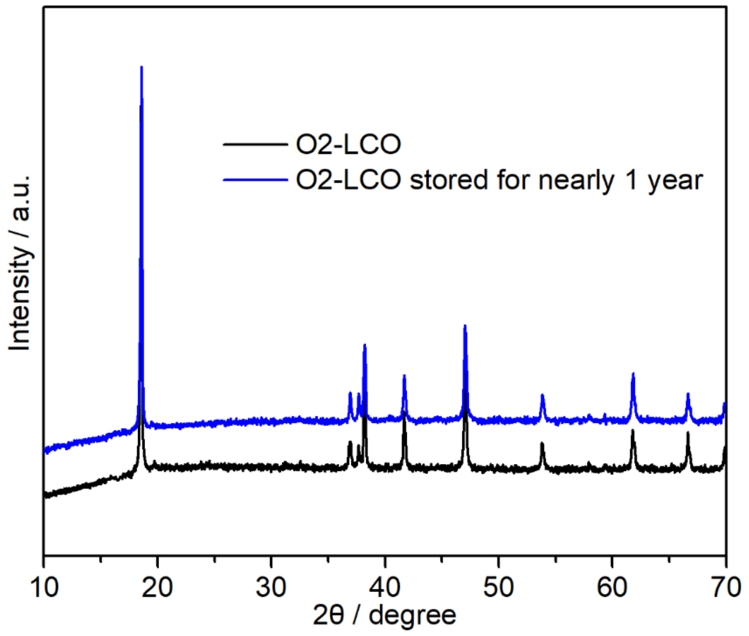

Figure S2. XRD patterns of fresh O2-LCO and old O2-LCO stored for nearly 1 year.

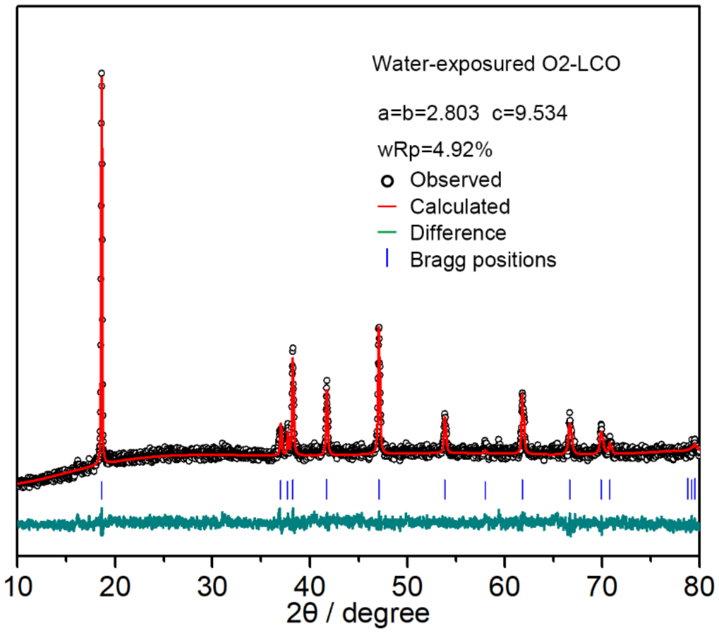

Figure S3. XRD patterns and Rietveld-refined results of O2-LCO after water-exposure.

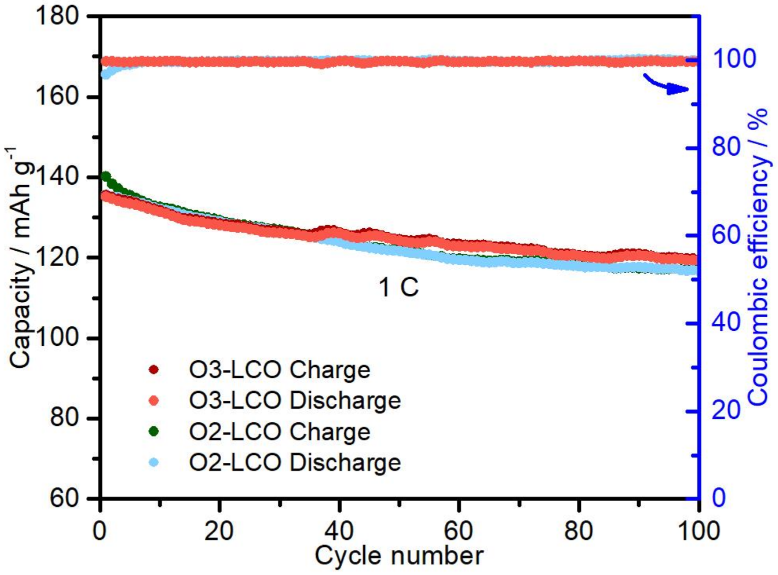

Figure S4. The cycle performance of the O3-LCO and O2-LCO electrodes in organic electrolyte.

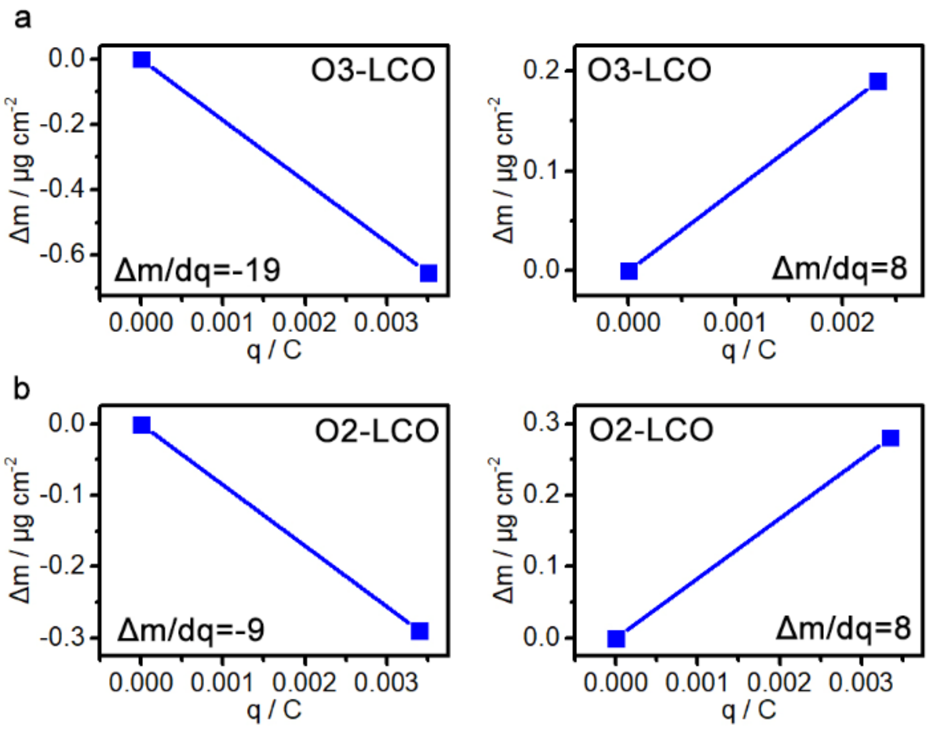

Figure S5. Electrode mass changes of the (a) O3-LCO and (b) O2-LCO electrodes during charge/discharge in the 1 M Li_2SO_4 aqueous electrolyte.

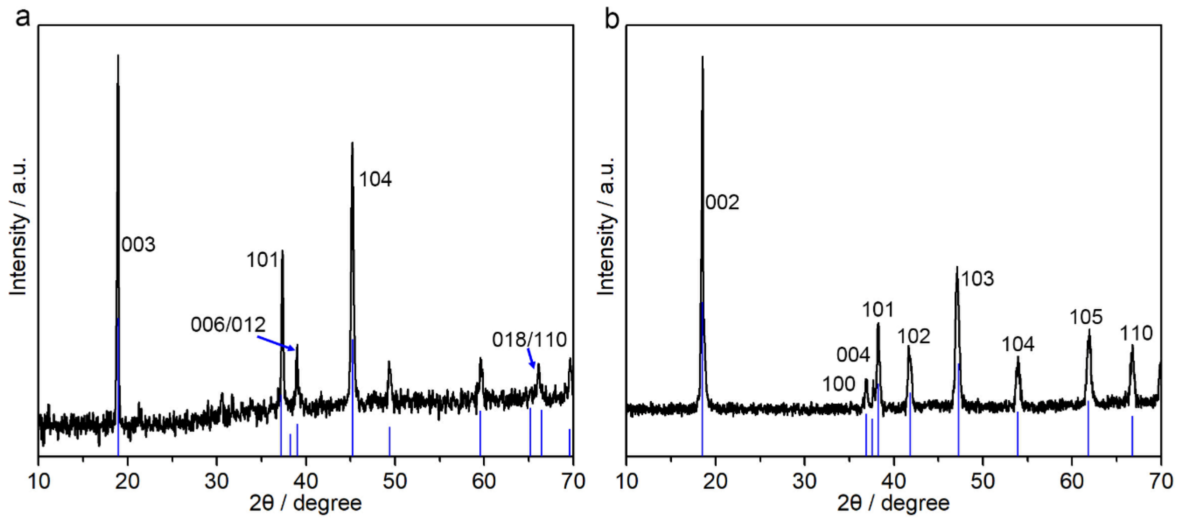

Figure S6. XRD patterns of O3-LCO and O2-LCO after 100 cycles.

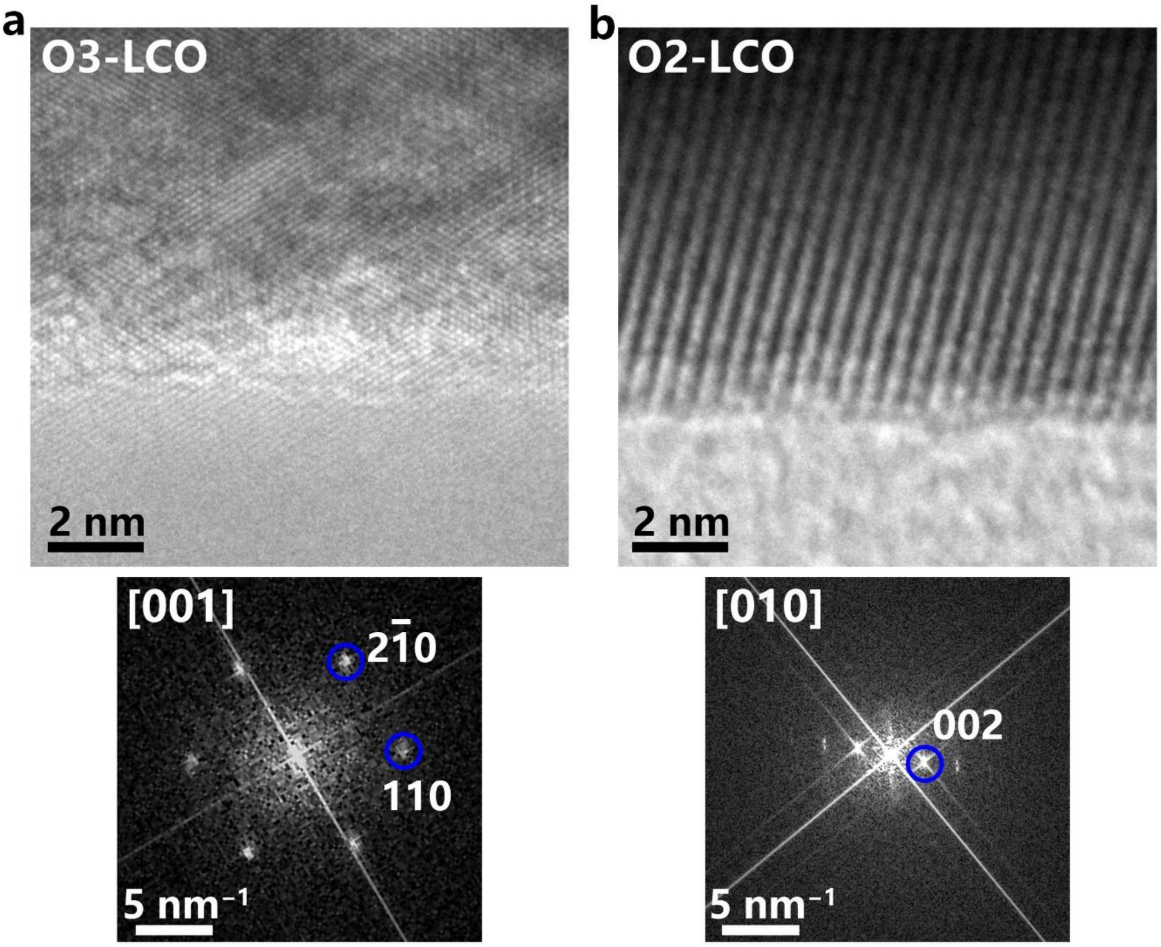

Figure S7. HRTEM images and corresponding FFT images of pristine O3-LCO and O2-LCO samples.

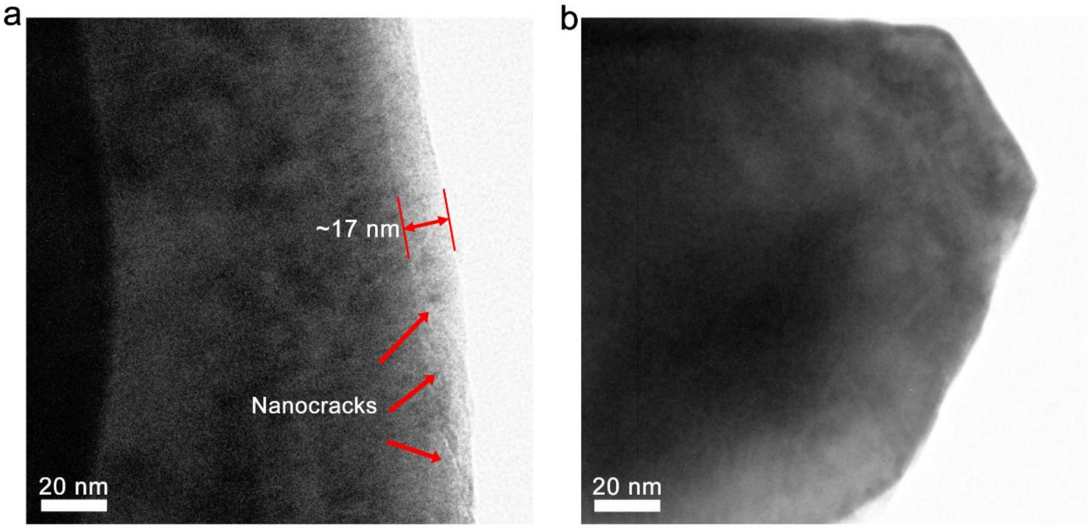

Figure S8. HRTEM images of (a) O3-LCO and (b) O2-LCO after 100 cycles in the 1 M Li_2SO_4 aqueous electrolyte.

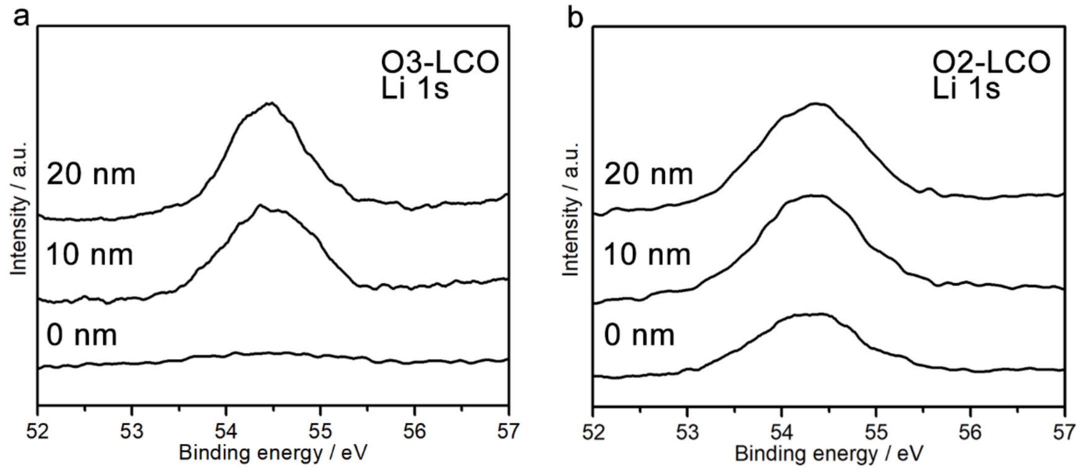

Figure S9. Depth XPS analysis of Li 1s of (a) O3-LCO and (b) O2-LCO electrodes after 100 cycles in the 1 M Li_2SO_4 aqueous electrolyte.

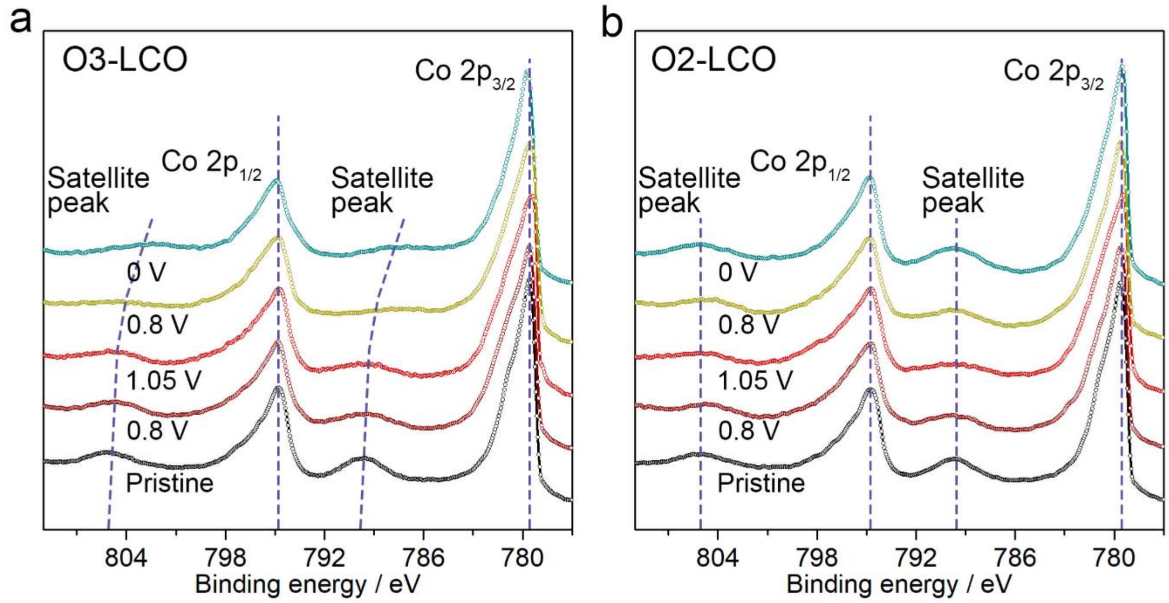

Figure S10. Ex situ Co 2p XPS spectra of (a) O3-LCO and (b) O2-LCO during the first charge/discharge processes in aqueous electrolyte.

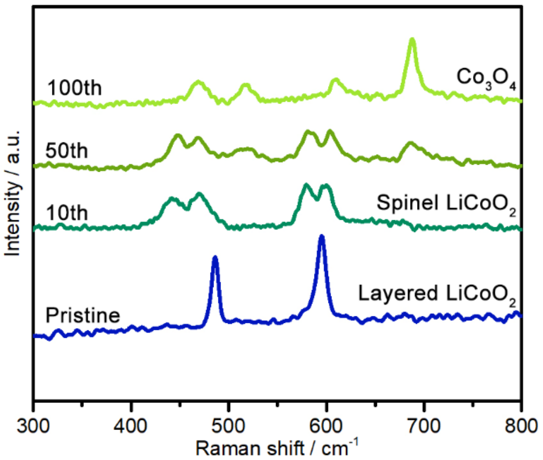

Figure S11. Raman spectra of O3-LCO with different cycle numbers in the pH of 7 aqueous electrolyte.

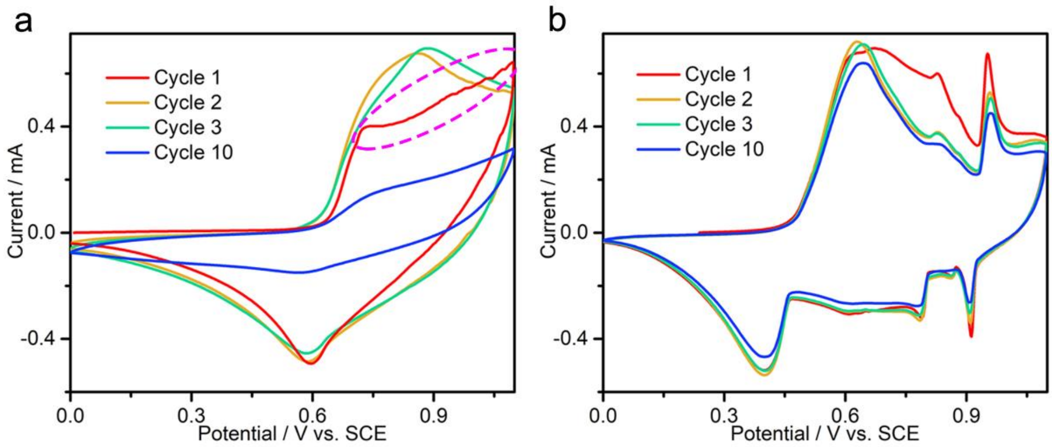

Figure S12. CV curves of the (a) O3-LCO and (b) O2-LCO electrodes in the 1 M Li_2SO_4 aqueous electrolyte.

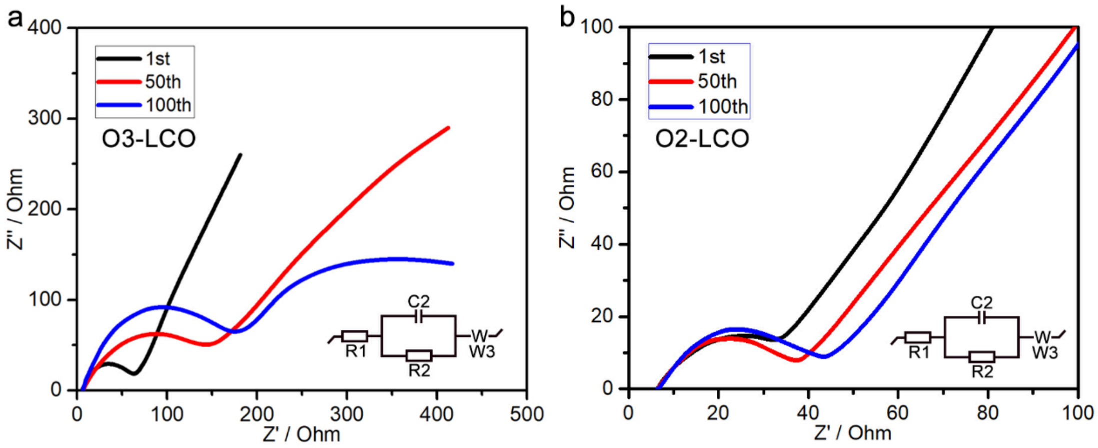

Figure S13. EIS spectra of the O3-LCO and O2-LCO electrodes at different cycle numbers in the 1 M Li_2SO_4 aqueous electrolyte.

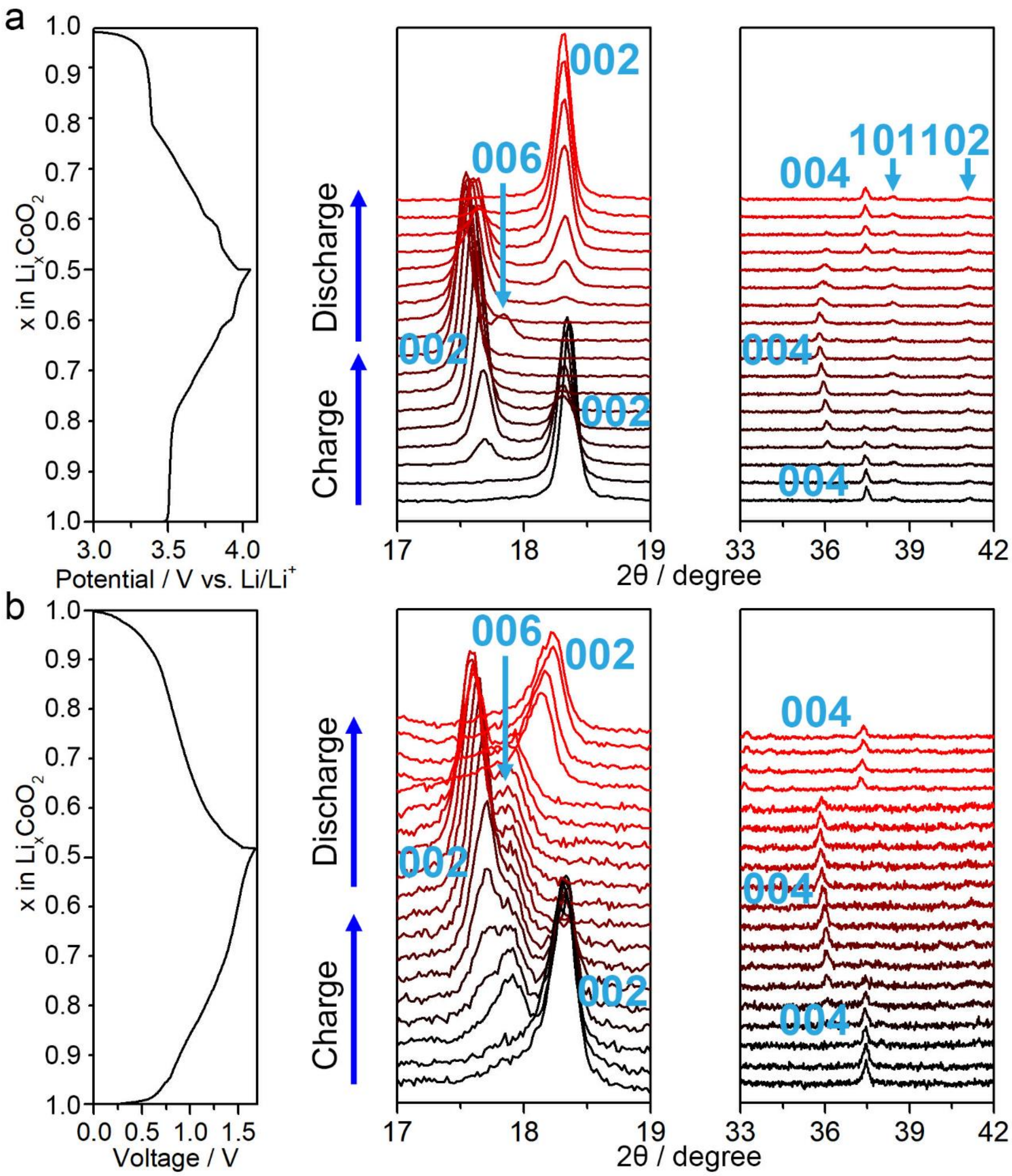

Figure S14. In situ XRD spectra of O2-LCO during charge/discharge in (a) organic and (b) aqueous electrolytes.

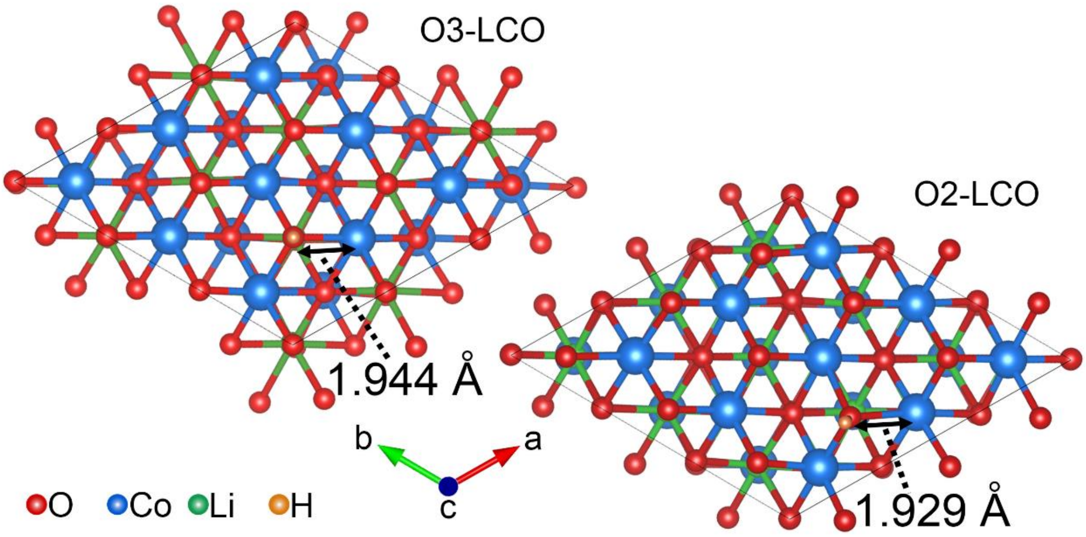

Figure S15. Supercell models for O3-LCO and O2-LCO with one H-O bond from the *c* direction.

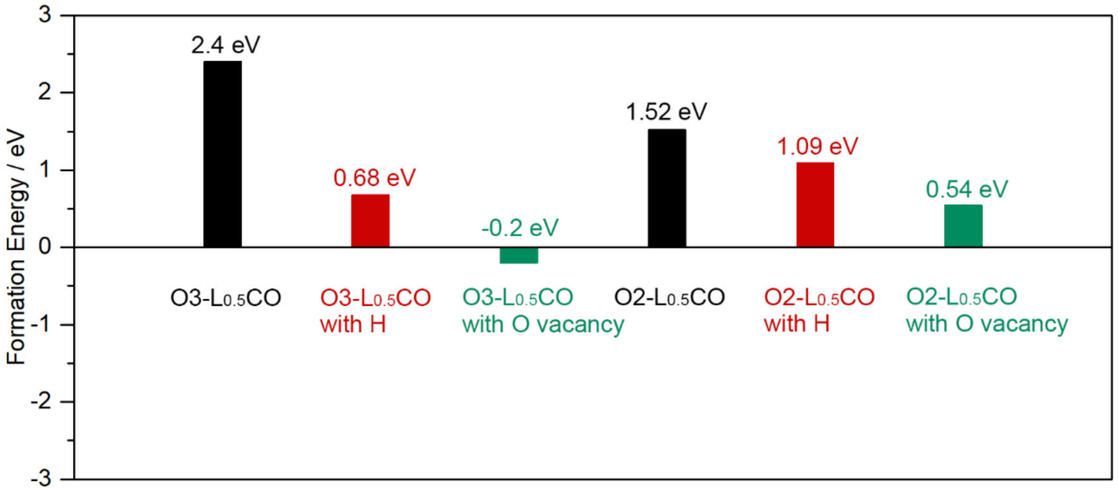

Figure S16. Co vacancy formation energies of O3-$L_{0.5}$CO and O2-$L_{0.5}$CO with and without H and O vacancies at half-delithiated state.

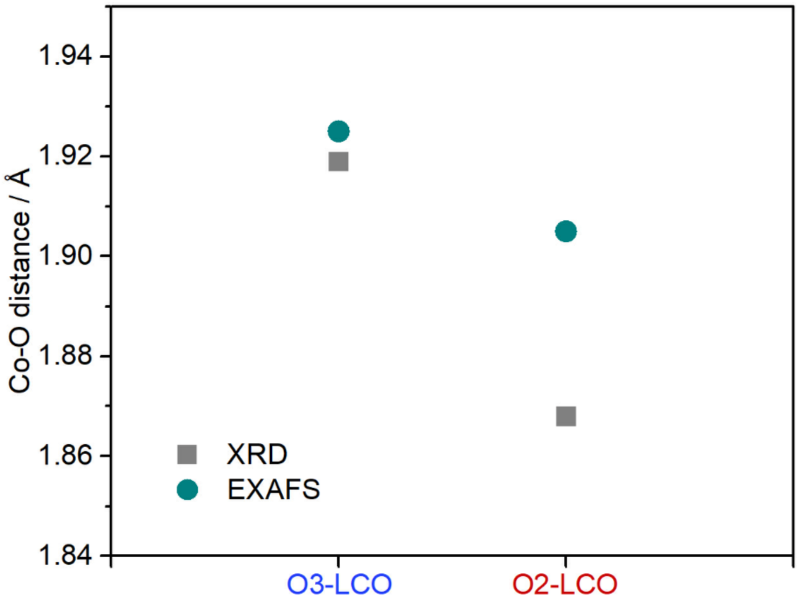

Figure S17. Co-O bond lengths of O3-LCO and O2-LCO obtained from XRD and EXAFS.

Table S1. Curve-fitting results for the Co K-edge EXAFS of O3-LCO and O2-LCO.

Sample	Shell	C.N.	R [Å]	σ^2 [10^{-2} Å^2]	R [%]
O3-LCO	Co-O	6	1.925	0.32	1.23
	Co-Co	6	2.815	0.28	
O2-LCO	Co-O	6	1.905	0.28	1.23
	Co-Co	6	2.803	0.26	
